# Effects of Elevated Ozone Levels on Photosynthesis, Biomass and Non-structural Carbohydrates of *Phoebe bournei* and *Phoebe zhennan* in Subtropical China

**DOI:** 10.3389/fpls.2018.01764

**Published:** 2018-11-30

**Authors:** Zhan Chen, Jixin Cao, Hao Yu, He Shang

**Affiliations:** Key Laboratory of Forest Ecology and Environment, State Forestry Administration, Research Institute of Forest Ecology, Environment and Protection, Chinese Academy of Forestry, Beijing, China

**Keywords:** ozone, *Phoebe bournei*, *Phoebe zhennan*, photosynthesis, non-structural carbohydrate

## Abstract

To assess the impacts of ozone (O_3_) on carbon metabolism of subtropical broadleaved tree species, seedlings of *Phoebe bournei* and *Phoebe zhennan* were exposed to elevated O_3_ levels in open-top chambers (OTCs) from June to November 2014. Three treatments were conducted in nine total OTCs, including charcoal-filter air (CF) as a control treatment, low O_3_ treatment ‘O3-1’ (∼100 nl l^-1^), and high O_3_ treatment ‘O3-2’ (∼150 nl l^-1^). Our findings demonstrated that elevated O_3_ levels significantly decreased the net photosynthesis rates (*P_n_*) and leaf, root, and total biomass of both species, while it did not significantly affect the root/shoot ratio in *P. bournei* and *P. zhennan*. O3-1 treatments significantly increased water soluble carbohydrates (WSC) in leaves of both tree species, while only increased the total non-structural carbohydrates (TNC) and starch in leaves of *P. bournei*; effects on *P. zhennan* were equivalent in comparison to the control treatment (CF). Likewise, there was no effect of treatment on the polysaccharide content of both tree species. The contents of polysaccharide, starch contents in fine roots of both species, and TNC in fine roots of *P. bournei* increased significantly in O3-1 compared to CF. O3-2 treatment significantly decreased starch and TNC in the fine roots of *P. bournei*, and significantly decreased polysaccharide, starch, WSC, and TNC in the fine roots of *P. zhennan*. Elevated O_3_ had no effects on leaf polysaccharide in both species, but O3-1 significantly increased polysaccharide in the fine roots of both species, and O3-1 significantly increased WSC in the leaves while decreased that in the fine roots of both species. These results suggested that elevated O_3_ levels have significant impacts on the carbon metabolism of both tree species in our study, with differential responses between tree species and among leaves and roots.

## Introduction

Ground-level ozone (O_3_) is potentially the most phytotoxic air pollutant for vegetation (Degl’Innocenti et al., 2002; Bytnerowicz et al., 2003), especially trees in forested ecosystems (Matyssek and Sandermann, 2003; Gerosa et al., 2015; Marco et al., 2015). There have been large increases in O_3_ levels since the pre-industrial times; global O_3_levels are predicted to either remain high or increase, particularly in rapidly developing countries such as China (Matyssek et al., 2010; Wang et al., 2012). Accelerated urbanization and industrial development in China leads to a substantial increase in tropospheric O_3_ emissions, with the mean daily O_3_ concentration in some regions reaching more than 50 nl l^-1^ during the growing season (Zhao et al., 2009; Tang et al., 2013). Consequently, the frequency of O_3_ pollution events in the lower troposphere has been observed to increase during the photochemically active seasons in these developing areas, which suggested significant deleterious effects of O_3_ elevation on regional air quality (Gao et al., 2005; Tie et al., 2009; Dufour et al., 2010).

The negative effects of O_3_ on vegetation have been known for more than 50 years (Köllner and Ghm, 2000). In vegetable farms, O_3_ has been shown to increase both the enzyme activity associated with general plant defense mechanisms and the antioxidant concentration (Caregnato et al., 2013; Kumari et al., 2015). However, only within the past 20 years, has O_3_ been considered a serious concern over to vegetation in China, especially to trees in forest ecosystems (Chen et al., 2015). It has been reported that elevated O_3_ not only induces visible tissue injury, inhibits photosynthesis, reduces plant biomass and crop yields but also alters belowground C-allocation and soil microbial community composition and diversity (Calatayud et al., 2004; Wang et al., 2007, 2015; Chen et al., 2008, 2014b; Zhang et al., 2012; Feng et al., 2014). Plant responses to O_3_ are complex and specific. Elevated O_3_ could increase both the enzyme activity associated with general plant defense mechanisms and the antioxidant concentration (Keutgen et al., 2005; Caregnato et al., 2013; Kumari et al., 2015).

Meanwhile, the drastic effects of O_3_ on the central processes of carbon (C) metabolism are well known, including the synthesis of photosynthetic carbohydrates and C-allocation (Mikkelsen, 1995; Andersen, 2003). However, the balance of structural and non-structural carbohydrates is also changed by O_3_ stress, which is one of the most intriguing biochemical responses of plants to O_3_ (Darrall, 1989; Chen et al., 2015). An important link between carbon dioxide (CO_2_) fixation and biomass production is formed through carbohydrate metabolism. Consequently, some studies showed changes in carbohydrate pools (Matyssek et al., 2010; Neufeld et al., 2012), carbohydrate metabolism (Sild et al., 2002) or allocation (Kleiner et al., 1999; Chen et al., 2014a) in response to elevated O_3_. However, few studies have focused on the effects of elevated O_3_ on carbon metabolism of tree species, especially in China.

Agathokleous et al. (2015) reviewed the effects of tropospheric O_3_ on wild plants, who found the study of the responses to O_3_ of endangered or threatened species to be indispensable. Some endangered or threatened species will likely be at risk of extinction as O_3_ concentrations increase. For this reason, research on plant species that are characterized as ‘endangered’ or ‘threatened’ will be essential for preserving biodiversity (Agathokleous et al., 2015). Some findings of O_3_ elevation on subtropical Chinese trees were reported including visible injury, photosynthesis decline and growth inhibition (*Liriodendron chinense*: Zhang et al., 2011; *Metasequoia glyptostroboides*: Feng et al., 2008; Zhang et al., 2014; *Cinnamomum camphora*: Feng et al., 2011; Cyclobalanopsisglauca: Zhang et al., 2013). However, there were less investigations focused on *Phoebe bournei* and *Phoebe zhennan* in response to elevated O_3_ in subtropical China, where we observed potentially harmful levels, with a maximum 8-h mean and peak O_3_ concentrations of 72.3 (8 October) and 97 nl l^-1^ (14:00, 16 October) in Taihe County, Jiangxi Province (Chen et al., 2015). Such current and future O_3_ levels may have adverse effects on carbon metabolism of subtropical tree species (Zhang et al., 2014) and definitely would affect the carbon balance of forested ecosystem in this region. Therefore, this study aims to examine the effects of elevated O_3_ on biomass, physiology, and non-structural carbohydrates by exposing seedlings of two Phoebe species to elevated O_3_ in open top chambers (OTCs) in subtropical China. *P. bournei* and *P. zhennan* are two important tree species in subtropical plantation; both tree species are on the national grade II list of rare and endangered plants. *P. bournei* and *P. zhennan* are formidable tree species in the *Lauraceae* family, existing at heights up to 20 or 30 m tall, respectively. Both species are endemic to China and threatened by habitat loss, thus these tree species are under second-class national protection in China.

In this study, *Phoebe* seedlings were planted in OTCs with charcoal filtered air or elevated O_3_ to investigate the effects of O_3_ on non-structural carbohydrates. Our specific hypotheses were that (1) O_3_ impacts carbon metabolism; (2) carbohydrates will differently respond to elevated O_3_ between *P. bournei* and *P. zhennan*; and that (3) elevated O_3_ would have different effects on carbohydrates in plant leaves and roots.

## Materials and Methods

### Experimental Site

The experimental site is located in the Qianyanzhou ecological station (115°03′29.2′′E, 26°44′29.1′′N) of the Chinese Academy of Sciences, which is a typical red earth hilly region in the mid-subtropical monsoon landscape zone of Taihe county, Jiangxi Province, China. The elevation is from 60 to 150 m, and the relative altitude ranges between 20 m and 50 m. According to meteorological data statistics monitored by the Qianyanzhou ecological station, the mean annual temperature, annual precipitation, annual evaporation was 17.8°C; 1471.2 mm; and 259.9 mm respectively. A detailed description of this study site was given in Chen et al. (2015).

### O_3_ Exposure

O_3_ fumigation began on June 25th and ended on November 12th, 2014. Experiments were carried out in OTCs, 2 m in diameter by 2.2 m in height. The boxes were connected with activated charcoal O_3_ decomposition filter (CF) and a fan, and run at two air changes per minute.

OTCs consist of an octagonal aluminum frame with a transparent film cover. O_3_ was distributed 80 cm above the canopy through a rotatable transparent pipe with many small holes (diameter of 10 mm at intervals of 10 cm), which released either CF air or O_3_+CF air, driven from a centrifugal blower. O_3_ was generated from pure oxygen by high-voltage electric discharge (Jinan SankangEnvi-tech Co., LTD., Shandong, China). O_3_ concentration in OTCs was regulated by mass flowmeters through controlling oxygen volume. The O_3_ concentrations within the OTCs were monitored by an O_3_ analyzer (49i, ThermoFisher). Nine OTCs were set in three lines and three rows, and the distance of OTCs was 4 m from each other.

Three treatments in this study included: CF, O3-1 and O3-2. In CF, the plants were exposed to CF air as the control treatment. Additional O_3_ was mixed with CF air to achieve elevated O_3_ concentrations (O3-1 and O3-2 treatments). The plants in both O_3_ treatments were exposed to O_3_ from 09:00 to 17:00. The accumulated exposure over a concentration threshold of 40 nl l^-1^ O_3_ based on hourly averages (AOT40, Fuhrer et al., 1997) was calculated. There were totally nine chambers with three replicates for each treatment.

### Growth Conditions

One-year-old nursery-grown container seedlings of *P. bournei* and *P. zhennan* were transplanted to flower pots (with diameter 20 cm and height 30 cm) with local red soil under ambient air conditions in April 2014. The soil pH was 5.36, organic matter content was 11.4 g⋅kg^-1^, and total nitrogen was 650.7 mg⋅kg^-1^. On June 5th, 2014, seedlings of similar height and basal diameter were selected for each species and randomly assigned to nine chambers with fifteen plants per OTC per species before O_3_ fumigation commenced. During the growing season, the seedlings were watered with tap water as required.

### Gas Exchange

Two upper fully expanded sun leaves per plant were randomly selected with three replicate plants per species in each chamber. The gas exchange was determined with a portable photosynthesis system fitted with a 6400–40 leaf chamber fluorimeter (LCF) (LI-6400, LI-COR Inc., Lincoln, NE, United States). Measurements were performed at ambient CO_2_ concentrations (34–360 ppm) at 50% RH. The block temperature was exposed to ambient air temperature. Photosynthetic activity at saturating light level was measured at 1000 μmol photons^-2^ s^-1^. All gas exchange measurements were conducted during 09:00–12:00 h.

### Biomass and Carbohydrate Analysis

Plants were harvested on the November 12th, 2014, when the growing season finishes in subtropical China. Five plants per species were randomly collected in each OTC and the leaves, stem and fine roots sampled separately. Dry mass was determined after oven-drying at 70°C until it reached a constant mass. Leaves and fine roots were ground to a fine powder through a 2 mm sieve for carbohydrate determination as described in Cao et al. (2017). In order to extract soluble sugars, we mixed 0.5 g powder with 50 mL of distilled water and steamed the mixture at high pressure for 2 h; subsequently, we filtered and diluted the mixture until it reached a constant volume. Starch was extracted with 10mL distilled water and 1 mL hydrochloric acid (2:1) in 0.1 g powder, then within a 100°C water bath for 8 h. After cooling to room temperature, NaOH was added to the mixture until it reached a neutral pH; then it was filtered and diluted to a constant volume. Both soluble sugar and starch were determined by injecting a 10 μl sample volume into an HPLC system (Agilent Technologies) using a sugar-park 1 chromatographic column (Waters, United States) and a refractive index detector (Waters HPLC 2695, Milford, MA, United States). Column temperature was 70°C and distilled water was used as the mobile phase (flow rate 0.6 ml⋅min^-1^). Carbohydrates are presented as fructose, glucose, polysaccharide, starch (the sum of residual starch and maltodextrins), water-soluble carbohydrates (WSC) (the sum of glucose, fructose, and sucrose), and TNCs (the sum of starch, polysaccharides, and WSC).

### Statistical Analysis

Treatment means were statistically compared using the statistical package SPSS (SPSS Inc., Chicago, IL, United States). One-way ANOVAs were used to determine differences in dry weights of various plant parts and carbohydrate contents among treatments. Two-factor analysis was used to assess the effects of O_3_ on biomass among plant species, and multiple-factor analysis was used to assess the effects of O_3_, species and organs on carbohydrates. Student-Newman-Keuls was calculated to determine whether there were significant differences between individual treatments; q-tests of the ANOVA were significant at *p* = 0.05.

## Results

### O_3_ Exposure

**FIGURE 1:**
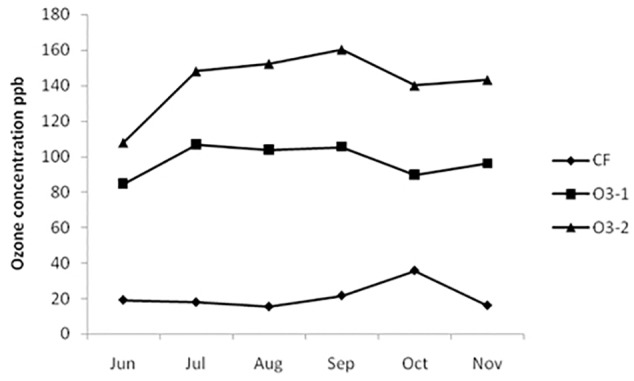
8-h mean O_3_ concentration of three treatments during the exposure period.

Mean 8 h O_3_ concentrations for CF, O3-1, and O3-2 treatments over the exposure periods are showed in Figure 1. The 8-h mean concentration was 21.0, 97.8, and 142.1 nl l^-1^ in CF, O3-1, and O3-2 treatments (Figure 1), and AOT40 values of 0.71, 54.5, and 96.2 ppm⋅h, respectively. The 8-h mean O_3_ concentration from 17:00–9:00 of the day was 5.74 nl/L.

### Biomass

Elevated O_3_ significantly affected dry biomass of *P. bournei and P. zhennan* seedlings except stem biomass. Compared with CF, leaf, roots and total biomass of *P. bournei* significantly decreased by 66.49%, 51.78, and 44.04% in O3-1 treatment, and 57.78%, 65.43%, and 51.69% in O3-2 treatment respectively. Relative to CF, leaf, root and total biomass of *P. zhennan* were reduced by 43.16%, 34.09%, and 42.53% in O3-1 treatment and 58.26%, 45.33%, and 50.48% in O3-2 treatment. No effects of O_3_ on stem biomass and root/shoot ratio were observed in either species (Table 1). Leaf biomass and root/shoot ratio showed significant difference between species, without interactive effect of O_3_ and species.

**Table 1:** Effects of ozone fumigation on biomass of both species (g dw).

species	Treatments	Leaf	Stem	Root	Total	Root/shoot ratio
*P.bournei*	CF	16.35 ± 1.12A	10.47 ± 2.50AB	7.64 ± 1.68A	35.52 ± 4.70A	0.27 ± 0.01BC
	O3-1	5.48 ± 0.06C	12.91 ± 2.43A	3.68 ± 0.51BC	19.88 ± 2.37BC	0.23 ± 0.01C
	O3-2	6.91 ± 1.06C	7.39 ± 1.38B	2.64 ± 0.13C	17.16 ± 3.21C	0.19 ± 0.03C
*P.zhennan*	CF	10.31 ± 1.21B	12.88 ± 0.88AB	7.67 ± 0.43A	31.22 ± 2.76AB	0.36 ± 0.06AB
	O3-1	5.86 ± 1.27C	7.86 ± 0.89AB	5.06 ± 0.65B	17.94 ± 3.40C	0.38 ± 0.02A
	O3-2	4.30 ± 0.28C	6.70 ± 0.27B	4.19 ± 0.12BC	15.46 ± 0.69C	0.38 ± 0.01A
O_3_		^∗∗^	ns	^∗∗^	^∗^	ns
Species		^∗^	ns	ns	ns	^∗∗^
O_3_^∗^Species		ns	ns	ns	ns	ns


*The values are means ± SE (n = 3). Results followed by different letters are statistically significant at the 0.05 level. ^∗∗^, significant at 0.01; ^∗^, significant at 0.05; ns, not significant.*

### Photosynthesis

**FIGURE 2:**
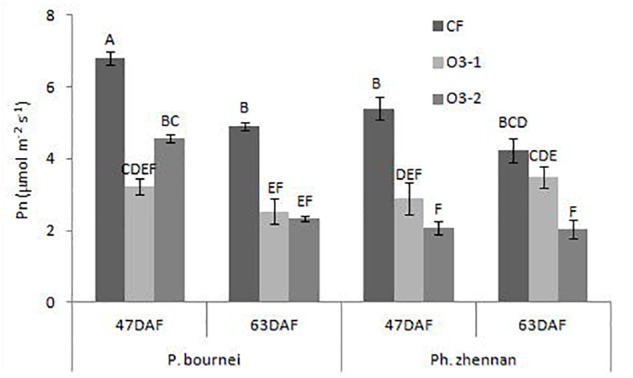
The change of photosynthesis rates (*P_n_*), in plants after exposure to different ozone concentrations. DAF, day after fumigation.

Both of the O_3_ treatments significantly decreased Pn of *P. bournei* at 47DAF (day after fumigation) and 63 DAF. For *P. zhennan*, significant reductions in Pn were observed under both O_3_ treatments in 47DAF and under O3-2 in 63 DAF (Figure 2). Averaged across the two measurements, O3-1 reduced Pn by 48.78 and 35.90%, and O3-2 reduced Pn by 42.89 and 54.73% in *P. bournei* and *P. zhennan*, respectively.

### Non-structural Carbohydrates

Elevated O_3_ had no effects on polysaccharide content in the leaves of either plant: *P. bournei* or *P. zhennan*. In roots, the polysaccharide contents in both species increased significantly under O3-1 treatments. However, when higher amounts of O_3_ (O3-2) were applied, only the polysaccharide concentration in *P. zhenan* roots decreased, as compared to CF. Starch content and both WSC and TNC concentrations in leaves of *P. bournei* were significantly higher in O3-1 treatment, as were WSC in leaves of *P. zhennan* in comparison to the control treatment (CF). O3-2 treatment significantly decreased WSC and TNC within leaves of *P. bournei*, as compared with CF. The TNC in fine roots of *P. bournei* and *P. zhennan* was mainly comprised of polysaccharides and starch (Table 2). Both elevated O_3_ treatments significantly decreased WSC in fine roots of *P. bournei* and *P. zhennan* comparing to that of CF. O3-1 treatment significantly increased polysaccharides, starch and TNC, and O3-2 treatment decreased polysaccharide and starch concentrations and decreased TNC compared to CF, with the exception of polysaccharides in fine root of *P. bournei* (Table 2). O_3_, species and plant organ had significant effects on polysaccharide, starch, WSC and TNC except species on polysaccharide. O_3_ and species showed highly significant interactive effects on these indices except WSC, while O_3_ and organ had extremely significant interactive effects on all of these indices (Table 2). There were also highly significant interactive effects on polysaccharide and TNC among O_3_, species and organ (Table 2).

**Table 2:** Effects of ozone fumigation on carbohydrate concentrations of leaves and roots in both species (g/100g dw).

Organ	Species	Treatments	Polysaccharide	Starch	WSC	TNC
Leaf	*P.bournei*	CF	5.32 ± 0.10CD	2.20 ± 0.15EF	6.37 ± 0.19B	13.89 ± 0.15C
		O3-1	5.26 ± 0.31CD	3.07 ± 0.12CD	7.46 ± 0.30A	15.79 ± 0.26B
		O3-2	5.90 ± 0.28C	1.82 ± 0.08F	4.42 ± 0.26D	12.15 ± 0.20DE
	*P.zhennan*	CF	5.73 ± 0.37C	2.10 ± 0.33EF	3.88 ± 0.14D	11.76 ± 0.59DE
		O3-1	5.74 ± 0.20C	1.83 ± 0.13F	5.27 ± 0.32C	12.56 ± 0.24D
		O3-2	5.90 ± 0.28C	1.82 ± 0.08F	4.42 ± 0.26D	12.15 ± 0.20DE
Root	*P.bournei*	CF	4.24 ± 0.04E	3.41 ± 0.22C	2.71 ± 0.37E	10.44 ± 0.15F
		O3-1	11.43 ± 0.31A	5.73 ± 0.09A	0.53 ± 0.14G	17.70 ± 0.08A
		O3-2	4.28 ± 0.18E	2.64 ± 0.48DE	1.71 ± 0.05F	8.64 ± 0.61G
	P.zhennan	CF	5.93 ± 0.18C	3.28 ± 0.03C	1.81 ± 0.07F	10.62 ± 0.44EF
		O3-1	6.87 ± 0.02B	4.40 ± 0.08B	0.38 ± 0.06G	11.50 ± 0.13E
		O3-2	4.76 ± 0.22DE	1.21 ± 0.10G	0.88 ± 0.02G	7.57 ± 0.33H
O_3_			^∗∗∗^	^∗∗∗^	^∗∗∗^	^∗∗∗^
Species			ns	^∗∗∗^	^∗∗∗^	^∗∗∗^
Organ			^∗∗∗^	^∗∗∗^	^∗∗∗^	^∗∗∗^
O_3_^∗^Species			^∗∗∗^	^∗∗∗^	ns	^∗∗∗^
O_3_^∗^Organ			^∗∗∗^	^∗∗∗^	^∗∗∗^	^∗∗∗^
Species^∗^Organ			^∗∗∗^	ns	^∗∗∗^	ns
O_3_^∗^Species^∗^Organ			^∗∗∗^	ns	ns	^∗∗∗^


*WSC, water-soluble carbohydrates; TNC, total non-structural carbohydrates. The values are means ± SE (n = 3). Results followed by different letters are statistically significant at the 0.05 level. ^∗∗∗^, significant at 0.001; ns, not significant.*

**FIGURE 3:**
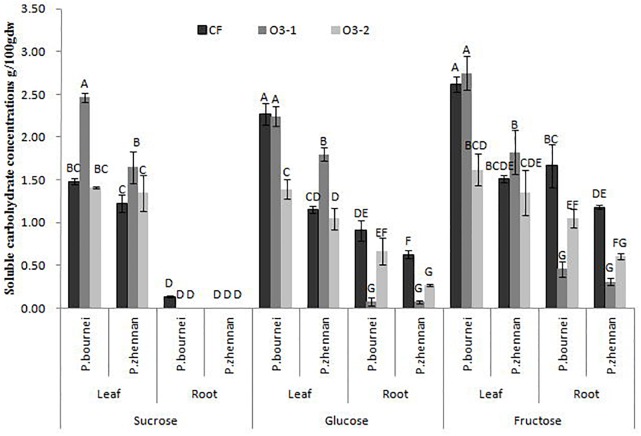
Effects of ozone fumigation on soluble carbohydrate concentrations of leaves and fine roots in both species (g⋅100g^-1^ dw).

Amongst WSC, only the high O_3_ treatment (O3-2) significantly decreased glucose and fructose level in leaves of *P. bournei*. O3-1 treatment increased the sucrose concentration, while O3-2 had no effect on sucrose, in comparison to CF levels (Figure 3). The increase in O_3_ concentration reduced the content of both glucose and fructose in fine roots of both tree species except for glucose of *P. bournei*under O3-2 (Figure 3), however, as the O_3_ concentration increased further, the decrease became smaller, i.e., O3-2-induced decrease was less than that by O3-1. O_3_, species and organ each individually impacted the content of sucrose, glucose and fructose (Table 3). O_3_ and species had significant interactive effects on sucrose and glucose but not fructose, which indicated that O_3_ had different effects on the content of both sucrose and glucose, excluding fructose, between *P. bournei* and *P. zhennan*. O_3_ and organ had significant interactive effects on sucrose, glucose and fructose. And the interactive effects of O_3_, species and organ were also significant for both sucrose and glucose, but not for fructose.

**Table 3:** Multi-factor analysis of O_3_, species and organ on soluble carbohydrate concentrations.

	Sucrose	Glucose	Fructose
O_3_	^∗∗∗^	^∗∗∗^	^∗∗∗^
Species	^∗∗∗^	^∗∗∗^	^∗∗∗^
Organ	^∗∗∗^	^∗∗∗^	^∗∗∗^
O_3_^∗^Species	^∗^	^∗∗^	ns
O_3_^∗^Organ	^∗∗^	^∗∗∗^	^∗^
Species^∗^Organ	^∗∗∗^	^∗∗∗^	^∗∗∗^
O_3_^∗^Species^∗^Organ	^∗∗^	^∗^	ns


*^∗∗∗^, significant at 0.001; ^∗∗^, significant at 0.01; ^∗^, significant at 0.05; ns, not significant.*

## Discussion

The decline of photosynthetic rates resulted in lower biomass. Photosynthetic products includes structural and non-structural carbohydrates, where the former supports the biomass including lignin and cellulose, and the latter including sucrose, glucose, fructose, and starch is the energy of the plants. It is generally recognized that elevated O_3_ limited the growth and decreased tree biomass because of photosynthesis inhibition. Our results showed that elevated O_3_ significantly decreased leaf, root and total biomass of *P. bournei* and *P. zhennan*. The marked decrease of photosynthesis under ozone exposure should be responsible for the reduced biomass. These results are consistent with other previous studies (birch: Pääkkönen et al., 1996; aspen: Dickson et al., 2001). No significant differences were observed between O3-1 and O3-2 treatments in leaf, stem or root biomass. Thus, at relatively-high O_3_ (O3-1) concentration biomass has been reduced with respect to a previous atmosphere with lower O_3_ concentration, but no additional decrease would be expected with further increases in O_3_, at least up to 140 nL/L (O3-2). Whereas, this study showed 40–50% biomass reduction in broadleaved evergreen Phoebes by 6-month ozone exposure, which was much larger than the previously studied Chinese broadleaved evergreen tree species (Feng et al., 2011). The substantial reduction in biomass may be due to Phoebe species sensitivity to long-term exposure to high concentrations of ozone. In our previous study, ambient O_3_ affected carbohydrate metabolism in leaves and roots of *P. bournei*, which indicated that *P. bournei* was sensitive to O_3_ (Chen et al., 2015); these findings were confirmed by the present study. Our results showed that there were no significant interactive effects on biomass between O_3_ and species, which indicated that there was no different effect of O_3_ on biomass of neither *P. bournei* nor *P. zhennan*. The inter-species distinction of biomass in response to O_3_ was not found in the Phoebe species in the present study, which is unlike findings from some studies, such as Hoshika et al. (2013), which found that ambient O_3_ had differential effects on biomass and biomass allocation between white birch and mountain birch.

As hypothesized, as the energy source of plants, elevated O_3_ levels impacted the non-structural carbohydrate profile in the leaves and fine roots of both *P. bournei* and *P. zhennan.* There were significant interactive effects on carbohydrates between O_3_ and species, or likewise between O_3_ and organs, which indicated that non-structural carbohydrates responded differently to elevated O_3_ in *P. bournei* and *P. zhennan*. Moreover, O_3_ had different effects on carbohydrates between leaves and roots. Overall, these findings provide support for all three of our hypotheses.

Carbohydrate metabolism is the major metabolic pathway in plants (Xing et al., 2015), with plant carbohydrates serving a major functional role as compatible solutes, which include hexoses, disaccharides, sugar alcohols, and complex sugars, all of which accumulate during stress (Jouve et al., 2004; Morsy et al., 2007). In this study, O3-1 treatment increased TNC in leaves of *P. bournei* and *P. zhennan*, which was consistent with increased TNC concentrations in response to elevated O_3_, while with reduction of biomass. In a Swedish open-top chamber experiment, the flag leaves of field-grown spring wheat in O_3_ treatment contained significantly more TNC than the flag leaves of the other treatments (Sild et al., 2002). These results suggest that although photosynthesis is inhibited resulting in biomass reduction, the energy source is stimulated to support the growth of plants under stress, and hence C-reserves can accumulate under moderate stress, which does not exceed levels shown to constrain C-acquisition (Palacio et al., 2007). Like in the present study TNC in *P. bournei* increased in O3-1 but decreased in O3-2 treatment, showing that O3-1 was likely not severe enough, while O3-2 may have been beyond the threshold, leading to constraints on C-acquisition. Increased TNC, including WSC in leaves in O3-1 treatment, may reserve energy for growth and development under less severe O_3_ stress, which may act as a protection mechanism.

Our finding that leaf starch content in *P. bournei* increased in the O3-1 treatment, was corroborated by other researchers (Günthardt-Goerg et al., 1993; Wellburn and Wellburn, 1994; Samuelson and Kelly, 1996; Zheng et al., 2000). Increased starch content indicates that the transport of photoassimilates out of the leaves may be reduced or inhibited (Landolt et al., 1994), acting as a carbon store for respiration under O_3_ stress (Palacio et al., 2007). We found that there was no significant effects of O_3_ on leaf starch in *P. zhennan*, which is consistent with some results from our another study, such that elevated O_3_ did not affect leaf starch of *Taxus wallichiana* (Cao et al., 2017). The responses of starch to elevated O_3_ differ among tree species, which is probably due to different sensitivities to O_3_ when the other environmental factors are remain changed, such as age, nutrition, or microclimate.

Sucrose is the primary source of carbon and energy, which is formed through a series of metabolic processes (Xing et al., 2015). Moreover, sucrose is an easily metabolized reducing sugar, which may serve as an immediate energy source upon stress removal (Morsy et al., 2007). In the present study, the leaves of plants exposed to O3-1 significantly accumulated sucrose, which supplies energy for repairing injuries resulting from O_3_ exposure. However, when O_3_ concentrations are above a specific threshold, plants lose their functional energy reserve by increasing sucrose, which is illustrated by our finding of no effects of O3-2 on sucrose, as compared to CF. O3-1 did not affect either glucose or fructose in *P. bournei* leaves, while the same treatments increased the glucose content in *P. zhennan* leaves. O3-2 treatment significantly reduced glucose and fructose contents in *P. bournei* leaves, but it had no effect on *P. zhennan* leaves, as compared to CF, which may have resulted from excessive O_3_ concentrations in the O3-2 treatment. The differential response of glucose and fructose in leaves among plant species may be due to inter-specific variation in responses to O_3._ With respect to O_3_ effects on different fractions of water soluble carbohydrate contents, different effects were observed depending on O_3_ concentration, each carbohydrate component, and species (Thomas et al., 2006; Chen et al., 2015; Cao et al., 2017), as well as the same species under different O_3_ exposure regimes (Köllner and Ghm, 2000). Although the different responses of these plant species to elevated O_3_ is complex, our study suggests that there is no universal effect of O_3_ concentration on WSC.

O3-1 treatment significantly increased starch and TNC in roots of both species, as was confirmed by our another study, in which ambient O_3_ increased starch and TNC in *P. bournei* roots (Chen et al., 2015). The accumulation of starch in roots after O_3_ exposure may have been attributed to reduced mycorrhizal infection, resulting in inhibited hydrolysis of starch to soluble sugars. Slankis (1973) reported evidence that mycorrhizal auxins enhance hydrolysis of starch to soluble sugars, thus a reduction in mycorrhizal infection after O_3_ exposure (Pérez-Soba et al., 1995) should be reflected in the accumulation of root starch concentrations. Conversely O3-2 treatment markedly decreased starch and TNC of roots in both species, as compared to CF, which was consistent with our finding that root starch decreased when growing in O_3_-polluted environments (Grulke et al., 2001; Thomas et al., 2002). O_3_ concentration in O3-2 treatment was much higher than that in O3-1, which was beyond the self-regulation capacity of both *Phoebe* species to O_3_ stress, brought about a significant reduction of starch and TNC.

## Conclusion

In this study we examined the effect of elevated O_3_ levels on carbon metabolism including biomass, photosynthesis, and non-structural carbohydrate of two tree species in subtropical China. Elevated O_3_ significantly decreased P_n_ and limited biomass production of *P. bournei* and *P. zhennan*. O_3_ strongly affected the carbohydrate content of both leaves and fine roots of both *P. bournei* and *P. zhennan*. However, differential responses to elevated O_3_ were observed both between tree species and among carbohydrate compounds. Our results are helpful for elucidating how elevated O_3_ levels impact carbon metabolism of local tree species in subtropical China, especially under future projected increases in atmospheric O_3_ concentrations. In the present study, seedlings were exposed for one growing season; for perennial trees a longer duration of exposure may be required for determining the effects of exposure. Further studies are needed to determine the physiological mechanisms of carbon metabolic changes, which will be valuable for predicting responses of other plant functional groups to prolonged O_3_ exposure.

## Author Contributions

ZC measured the carbohydrates, analyzed the data, and wrote the manuscript. JC completed the field experiments. HS designed the whole project. HY measured the biomass and photosynthesis.

## Conflict of Interest Statement

The authors declare that the research was conducted in the absence of any commercial or financial relationships that could be construed as a potential conflict of interest.

## References

[B1] Agathokleous E. Saitanis C. J. Koike T. (2015). Tropospheric O3, the nightmare of wild plants: a review study. *J. Agric. Meteorol.* 71 142–152. 10.2480/agrmet.d-14-00008

[B2] Andersen C. P. (2003). Source–sink balance and carbon allocation below ground in plants exposed to ozone. *New Phytol.* 157 213–228. 10.1046/j.1469-8137.2003.00674.x 33873636

[B3] Bytnerowicz A. Arbaugh M. J. Alonso R. (2003). “Ozone air pollution in the Sierra Nevada: distribution and effects on forests,” in *Developments in Environmental Science* Vol. 2 ed. Krupa S. V. (Amsterdam: Elsevier), 402.

[B4] Calatayud A. Iglesias D. J. Talón M. Barreno E. (2004). Response of spinach leaves (*Spinacia oleracea* L.) to ozone measured by gas exchange, chlorophyll a fluorescence, antioxidant systems, and lipid peroxidation. *Photosynthetica* 42 23–29. 10.1023/b:phot.0000040565.53844.c6

[B5] Cao J. Chen Z. Yu H. Shang H. (2017). Differential responses in non-structural carbohydrates of *Machilus ichangensis* rehd. etwils. and *Taxus wallichiana* zucc. var. chinensis (pilg.) florin seedlings to elevated ozone. *Forests* 8 323–335.

[B6] Caregnato F. F. Bortolin R. C. Junior A. M. D. Cláudio J. Moreira F. (2013). Exposure to elevated ozone levels differentially affects the antioxidant capacity and the redox homeostasis of two subtropical *Phaseolus vulgaris* L. varieties. *Chemosphere* 93 320–330. 10.1016/j.chemosphere.2013.04.084 23714146

[B7] Chen Z. Shang H. Cao J. Yu H. (2015). Effects of ambient ozone concentrations on contents of nonstructural carbohydrates in Phoebe bournei and *Pinus massoniana* seedlings in subtropical China. *Water Air Soil Pollut.* 226 310–317. 10.1007/s11270-015-2555-7

[B8] Chen Z. Wang X. Feng Z. Xiao Q. Duan X. (2008). Impact of elevated O3 on soil microbial community function under wheat crop. *Water Air Soil Poll.* 198 189–198. 10.1007/s11270-008-9838-1

[B9] Chen Z. Wang X. K. Shang H. (2014a). Ozone effects on soil microbial community of rice investigated by 13C isotope labeling (Chinese with English abstract). *Environ. Sci.* 35 3911–3917. 10.13227/j.hjkx.2014.10.038 25693401

[B10] Chen Z. Wang X. K. Shang H. (2014b). Using 13C isotope to investigate O3 effects on C fixation and translocation of rice (Chinese with English abstract). *Chin. J. Ecol.* 33 1983–1988.

[B11] Darrall N. (1989). The effect of air pollutants on physiological processes in plants. *Plant Cell Environ.* 12 1–30. 10.1111/j.1365-3040.1989.tb01913.x

[B12] Degl’Innocenti E. Guidi L. Soldatini G. F. (2002). Characterisation of the photosynthetic response of tobacco leaves to ozone: CO_2_ assimilation and chlorophyll fluorescence. *J. Plant Physiol.* 159 845–853. 10.1078/0176-1617-00519

[B13] Dickson R. E. Coleman M. D. Pechter P. Karnosky D. (2001). Growth and crown architecture of two aspen genotypes exposed to interacting ozone and carbon dioxide. *Environ. Pollut.* 115 319–334. 10.1016/s0269-7491(01)00225-1 11789916

[B14] Dufour G. Eremenko M. Orphal J. Flaud J. M. (2010). IASI observations of seasonal and day-to-day variations of tropospheric ozone over three highly populated areas of China: Beijing, Shanghai, and Hong Kong. *Atmos. Chem. Phys.* 10 3787–3801. 10.5194/acp-10-3787-2010

[B15] Feng Z. Sun J. Wan W. Hu E. Calatayud V. (2014). Evidence of widespread ozone-induced visible injury on plants in Beijing, China. *Environ. Pollut.* 193 296–301. 10.1016/j.envpol.2014.06.004 24989347

[B16] Feng Z. Z. Niu J. J. Zhang W. W. Wang X. K. Yao F. F. Tian Y. (2011). Effects of ozone exposure on sub-tropical evergreen *Cinnamomum camphora* seedlings grown in different nitrogen loads. *Trees* 25 617–625. 10.1007/s00468-011-0538-x

[B17] Feng Z. Z. Zheng H. Q. Wang X. K. Zheng Q. W. Feng Z. Z. (2008). Sensitivity of *Metasequoia glyptostroboides* to ozone stress. *Photosynthetica* 46 463–465. 10.1007/s11099-008-0079-8

[B18] Fuhrer J. Skarby L. Ashmore M. R. (1997). Critical levels for ozone effects on vegetation in Europe. *Environ. Pollut.* 97 91–106. 10.1016/s0269-7491(97)00067-5 15093382

[B19] Gao J. Wang T. Ding A. Liu C. (2005). Observational study of ozone and carbon monoxide at the summit of mount Tai (1534m a.s.l.) in central-eastern China. *Atmos. Environ.* 39 4779–4791. 10.1016/j.atmosenv.2005.04.030

[B20] Gerosa G. Fusaro L. Monga R. Finco A. Fares S. Manes F. (2015). A flux-based assessment of above and below ground biomass of Holm oak (*Quercus ilex* L.) seedlings after one season of exposure to high ozone concentrations. *Atmos. Environ.* 113 41–49. 10.1016/j.atmosenv.2015.04.066

[B21] Grulke N. W. Andersen C. P. Hogsett W. E. (2001). Seasonal changes in above- and belowground carbohydrate concentrations of *Ponderosa* pine along a pollution gradient. *Tree Physiol.* 21 173–181. 10.1093/treephys/21.2-3.173 11303648

[B22] Günthardt-Goerg M. Matyssek R. Scheidegger C. Keller T. (1993). Differentiation and structural decline in the leaves and bark of birch (*Betula pendula*) under low ozone concentrations. *Trees* 7 104–114. 10.1007/bf00225477

[B23] Hoshika Y. Tatsuda S. Watanabe M. Wang X. Watanabe Y. Saito H. (2013). Effect of ambient ozone at the somma of Lake Mashu on growth and leaf gas exchange in *Betula ermanii* and *Betula platyphylla* var. japonica. *Environ. Exp. Bot.* 90 12–16. 10.1016/j.envexpbot.2012.11.003

[B24] Jouve L. Hoffmann L. Hausman J. F. (2004). Polyamine, carbohydrate, and proline content changes during salt stress exposure of Aspen (*Populus tremula* L.): involvement of oxidation and osmoregulation metabolism. *Plant Biol.* 6 74–80. 10.1055/s-2003-44687 15095137

[B25] Keutgen A. J. Noga G. Pawelzik E. (2005). Cultivar-specific impairment of strawberry growth, photosynthesis, carbohydrate and nitrogen accumulation by ozone. *Environ. Exp. Bot.* 53 271–280. 10.1016/j.envexpbot.2004.04.003

[B26] Kleiner K. W. Raffa K. F. Dickson R. E. (1999). Partitioning of 14C-labeled photosynthate to allelochemicals and primary metabolites in source and sink leaves of aspen: evidence for secondary metabolite turnover. *Oecologia* 119 408–418. 10.1007/s004420050802 28307764

[B27] Köllner B. Ghm K. (2000). Changes in carbohydrates, leaf pigments and yield in potatoes induced by different ozone exposure regimes. *Agric. Ecosyst. Environ.* 78 149–158. 10.1016/s0167-8809(99)00118-8

[B28] Kumari S. Agrawal M. Singh A. (2015). Effects of ambient and elevated CO_2_ and ozone on physiological characteristics, antioxidative defense system and metabolites of potato in relation to ozone flux. *Environ. Exp. Bot.* 109 276–287. 10.1016/j.envexpbot.2014.06.015

[B29] Landolt W. Günthardt-Goerg M. Pfenninger I. Scheidegger C. (1994). Ozone-induced microscopical changes and quantitative carbohydrate contents of hybrid poplar (*Populus* × *euramericana*). *Trees* 8 183–190. 10.1007/bf00196845

[B30] Marco D. A. Sicard P. Vitale M. Carriero G. Renou C. Paoletti E. (2015). Metrics of ozone risk assessment for Southern European forests: canopy moisture conten as a potential plant response indicator. *Atmos. Environ.* 120 182–190. 10.1016/j.atmosenv.2015.08.071

[B31] Matyssek R. Sandermann H. (2003). Impact of ozone on trees: an ecophysiological perspective. *Prog. Bot.* 64 349–404. 10.1007/978-3-642-55819-1_15

[B32] Matyssek R. Wieser G. Ceulemans R. Rennenberg H. Pretzsch H. Haberer K. (2010). Enhanced ozone strongly reduces carbon sink strength of adult beech (*Fagus sylvatica*) – Resume from the free-air fumigation study at Kranzberg Forest. *Environ. Pollut.* 158 2527–2532. 10.1016/j.envpol.2010.05.009 20570421

[B33] Mikkelsen T. (1995). Physiological responses ni *Fagus sitvatica* L. exposed to low levels of ozone in open-top chambers. *Trees* 9 355–361. 10.1007/bf00202500

[B34] Morsy M. R. Jouve L. Hausman J. F. Hoffmann L. Stewart J. Mc. D. (2007). Alteration of oxidative and carbohydrate metabolism under abiotic stress in two rice (*Oryza sativa* L.) genotypes contrasting in chilling tolerance. *J. Plant Phys.* 164 157–167. 10.1016/j.jplph.2005.12.004 16500726

[B35] Neufeld H. S. Peoples S. J. Davison A. W. Chappelka A. H. Somers G. L. Thomley J. E. (2012). Ambient ozone effects on gas exchange and total non-structural carbohydrate levels in cutleaf condflower (*Rudbeckia laciniata* L.) growing in Great Smoky Mountains National Park. *Environ. Pollut.* 160 74–81. 10.1016/j.envpol.2011.09.010 22035928

[B36] Pääkkönen E. Vahala J. Holopainen T. Karjalainen R. Kärenlampi L. (1996). Growth responses and related biochemical and ultrastructural changes of the photosynthetic apparatus in birch (*Betula pendula*) saplings exposed to low concentrations of ozone. *Tree Physiol.* 16 597–605. 10.1093/treephys/16.7.597 14871697

[B37] Palacio S. Maestro M. Montserrat-Martí G. (2007). Seasonal dynamics of non-structural carbohydrates in two species of mediterranean sub-shrubs with different leaf phenology. *Environ. Exp. Bot.* 59 34–42. 10.1016/j.envexpbot.2005.10.003

[B38] Pérez-Soba M. Dueck T. A. Puppi G. Kuiper P. J. C. (1995). Interactions of elevated CO2, NH3 and O3 on mycorrhizal infection, gas exchange and N metabolism in saplings of *Scots pine*. *Plant Soil* 176 107–116. 10.1007/bf00017681

[B39] Samuelson L. J. Kelly J. M. (1996). Carbon partitioning and allocation in northern red oak seedlings and mature trees in response to ozone. *Tree Physiol.* 16 853–858. 10.1093/treephys/16.10.853 14871676

[B40] Sild E. Pleijel H. Selldén G. (2002). Elevated ozone (O3) alters carbohydrate metabolism during grain filling in wheat (*Triticum aestivum* L.). *Agric. Ecosyst. Environ.* 92 71–81. 10.1016/s0167-8809(01)00270-5

[B41] Slankis V. (1973). “Hormonarle lationshipins mycorrhizal development,” in *Ectomycorrhizae: Their Ecology and Physiology*, eds Marks G. C. Kozlowski T. T. (New York, NY: Academic Press), 231–298.

[B42] Tang H. Takigawa M. Liu G. Zhu J. Kobayashi K. (2013). A projection of ozone induced wheat production loss in China and India for the years 2000 and 2020 with exposure-based and flux-based approaches. *Glob. Change Biol.* 19 2739–2752. 10.1111/gcb.12252 23661338

[B43] Thomas V. F. D. Braun S. Flückiger W. (2006). Effects of simultaneous ozone exposure and nitrogen loads on carbohydrate concentrations, biomass, growth, and nutrient concentrations of young beech trees (*Fagus sylvatica*). *Environ. Pollut.* 143 341–354. 10.1016/j.envpol.2005.11.036 16458397

[B44] Thomas V. F. D. Hiltbrunner E. Braun S. Flückiger W. (2002). Changes in root starch contents of mature beech (*Fagus sylvatica* L.) along an ozone and nitrogen gradient in Switzerland. *Phyton* 42 223–228.

[B45] Tie X. Geng F. Peng L. Gao W. Zhao C. (2009). Measurement and modeling of O3 variability in Shanghai, China: application of the WRF-Chem model. *Atmos. Environ.* 43 4289–4302. 10.1016/j.atmosenv.2009.06.008

[B46] Wang X. Qu L. Mao Q. Watanabe M. Hoshika Y. Koyoma A. (2015). Ectomycorrhizal colonization and growth of the hybrid larch F1 under elevated CO_2_ and O_3_. *Environ. Pollut.* 197 116–126. 10.1016/j.envpol.2014.11.031 25521414

[B47] Wang X. K. Manning W. Feng Z. W. Zhu Y. G. (2007). Ground level ozone in China: distribution and effects on crop yields. *Environ. Pollut.* 147 394–400. 10.1016/j.envpol.2006.05.006 16973249

[B48] Wang Y. Konopka P. Liu H. Chen H. Müller R. Plöger F. (2012). Tropospheric ozone trend over beijing from 2002-2020: ozonesonde measurements and modeling analysis. *Atmos. Chem. Phys.* 12 11175–11199. 10.5194/acpd-12-11175-2012

[B49] Wellburn F. A. M. Wellburn A. R. (1994). Atmospheric ozone affects carbohydrate allocation and winter hardiness of *Pinus halepensis* Mill. *J. Exp. Bot.* 45 607–614. 10.1093/jxb/45.5.607

[B50] Xing W. Li L. Gao P. Li H. Shao Q. Shu S. (2015). Effects of grafting with pumpkin rootstock on carbohydrate metabolism in cucumber seedlings under Ca(NO3)2 stress. *Plant Physiol. Biol.* 87 124–132. 10.1016/j.plaphy.2014.12.011 25579659

[B51] Zhang W. Feng Z. Wang X. Niu J. (2012). Responses of native broadleaved woody species to elevated ozone in subtropical China. *Environ. Pollut.* 163 149–157. 10.1016/j.envpol.2011.12.035 22325443

[B52] Zhang W. W. Feng Z. Z. Wang X. K. Niu J. F. (2013). Elevated ozone negatively affects photosynthesis of current-year leaves but not previous-year leaves in evergreen *Cyclobalanopsis glauca* seedlings. *Environ. Pollut.* 184 676–681. 10.1016/j.envpol.2013.04.036 23714144

[B53] Zhang W. W. Feng Z. Z. Wang X. K. Niu J. F. (2014). Impacts of elevated ozone on growth and photosynthesis of *Metasequoia glyptostroboides* Hu et Cheng. *Plant Sci.* 226 182–188. 10.1016/j.plantsci.2014.06.005 25113463

[B54] Zhang W. W. Niu J. F. Wang X. K. Tian Y. Yao F. F. Feng Z. Z. (2011). Effects of ozone exposure on growth and photosynthesis of the seedlings of *Liriodendron chinense* (Hemsl.) Sarg, a native tree species of subtropical China. *Photosynthetica* 49 29–36. 10.1007/s11099-011-0003-5

[B55] Zhao C. Wang Y. Zeng T. (2009). East china plains: a “Basin” of ozone pollution. *Environ. Sci. Technol.* 43 1911–1915. 10.1021/es8027764 19368191

[B56] Zheng Y. Lyons T. Barnes J. (2000). Effects of ozone on the production and utilization of assimilates in *Plantago* major. *Environ. Exp. Bot.* 43 171–180. 10.1016/s0098-8472(99)00056-8

